# Phylogenetic analysis of the complete mitochondrial genome of *Anguilla japonica* (Anguilliformes, Anguillidae)

**DOI:** 10.1080/23802359.2018.1467225

**Published:** 2018-04-27

**Authors:** Kehua Zhu, Li Gong, Lihua Jiang, Liqin Liu, Zhenming Lü, Bing-jian Liu

**Affiliations:** a National engineering Laboratory of Marine Germplasm Resources Exploration and Utilization, Zhejiang Ocean University, Zhoushan, China;; b National engineering research center for facilitated marine aquaculture, Marine Science and Technology College, Zhejiang Ocean University, Zhoushan, China;; c Laboratory for Marine Fisheries Science and Food Production Processes, Qingdao National Laboratory for Marine Science and Technology, Qingdao, China

**Keywords:** *Anguilla japonica*, mitogenome, phylogeny

## Abstract

The Japanese eel (*Anguilla japonica*), which was listed as Endangered (EN) under the International Union for the Conservation of Nature due to the declines in its abundance, overfishing, and its narrow range distribution. To better protect this species, we described the complete mitogenome of *A. japonica* in this study. The mitogenome is 16717bp in length and contains 13PCGs, two rRNA genes (12S rRNA and 16S rRNA), 22 tRNA genes, and a putative control region (CR) and one origin of replication on the light-strand (OL). Moreover, the 13 PCGs encode 3813 amino acids in total, all the protein-coding genes use the initiation codon ATG except COI uses GTG. Most of them have TAA or TAG as the stop codon, except COI uses AGG and two PCGs (COII and ND4) use an incomplete stop codon T. A phylogenetic tree based on the Neighbour Joining method was constructed to provide relationship within *Anguilla*, which could be a useful basis for conservation of this species.

The *A. japonica* is a species of anguillid eel found in Japan, Korea, China, Vietnam, and northern Philippineshe (Tsukamoto 1992). According to the relevant studies, the Japanese eel population, along with anguillid eel populations worldwide, have declined drastically in recent years, presumably due to a combination of overfishing and habitat loss or changing water conditions in the ocean interfering with spawning and the transport of their leptocephali (Han et al. 2010). Therefore, conservation approaches for this species are desperately needed. In this study, we determined and described the complete mitochondrial genome of *A. japonica* and explored the phylogenetic relationship within *Anguilla*, which could be an effective geneticmarker for such purposes.

The specimen was collected from Fuchun River in Fuyang, China (27°56′17″N; 119°51′15″E) and stored in laboratory of Zhejiang Ocean University with accession number 20150826ML22. The complete mitogenome of *A. japonica* is 16717bp long (GeneBank Accession No. MH050933) and contains 13PCGs, 2 ribosomal RNA genes, 22 transfer RNA genes, and 2 main non-coding regions, this feature was similar to the typical mitogenome of other vertebrates (Miya et al. 2001). The overall base composition is 33.7% A, 25.9% C, 24.8% T and 15.6% G. Twelve PCGs, 14 tRNA genes and two rRNA genes were located on the heavy strand, while one PCG (ND6) and eight tRNA genes (Gln, Ala, Asn, Cys, Tyr, Ser, Glu and Pro) on the light strand. The 13 PCGs genes encode 3813 amino acids in total. All the protein-coding genes use the initiation codon ATG except COI uses GTG, which is quite common in vertebrate mtDNA (Behera et al. 2017; Jiang et al. 2018; Zhu et al. 2018). Most of them have TAA or TAG as the stop codon, except COI uses AGG, and two PCGs (COII and ND4) ended with a single T, these incomplete termination codons were presumably completed as TAA by post-transcriptional polyadenylation (Ojala et al. 1981). Eleven overlapping areas (46 bp in total) were observed, notable overlapping occurred at three pairs of PCGs. ATP8 and ATP6 overlapped by 10 nucleotides, ND4L and ND4 by 7 bp, and ND5 and ND6 (encoded on opposing stand) by 4 bp. The CR is determined to be 969 bp, which is located between the tRNA-Pro and tRNA-Phe genes, by comparing the sequences of the CR with that of other teleost, and the OL is located in a cluster of five tRNA genes as in other vertebrates (Boore 1999; Zhang et al. 2014; Gong et al. 2017), which has the potential to fold into a stable stem-loop secondary structure, with a stem formed by 12 paired nucleotides and a loop of 12 nucleotides.

We performed bootstrap analyses (1000 replicates) to evaluate relative levels of support for various nodes in the phylogenie (Figure 1). The NJ tree indicated two major groups of this genus and demonstrated that *A. japonica* has a closest relationship with *A. megastoma*, which are consistent with the results based on morphology and other molecular methods (Inoue et al. 2010).

**Figure 1.**
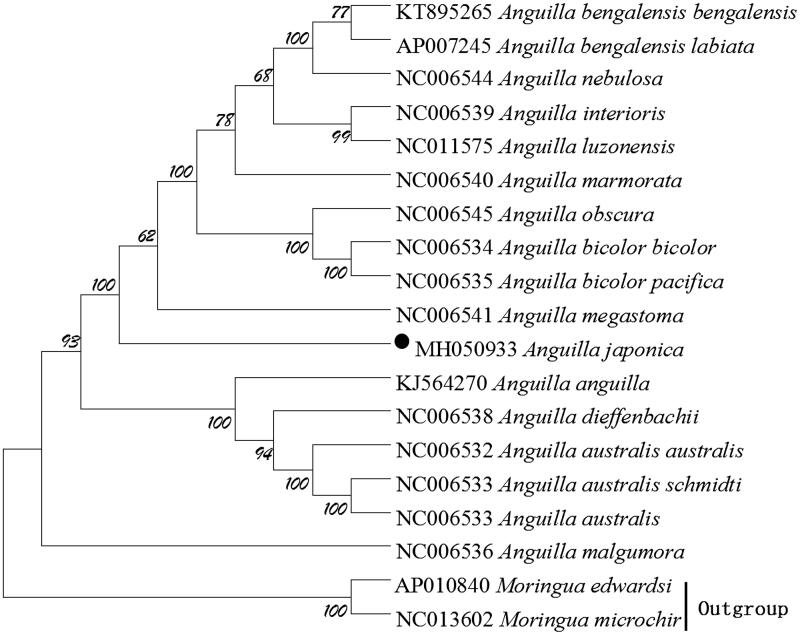
The phylogenetic tree of A. japonica and other 16 Anguilla species was constructed using the Neighbor Joining (NJ) methods based on 12 protein-coding genes encoded by the heavy strand. The bootstrap values are based on 1,000 resamplings and the number at each node is the bootstrap probability. The number before the species name is the GenBank accession number. The genome sequence in this study is labelled with a black spot.
